# Lupus aortitis mimicking an aortic intramural hematoma

**DOI:** 10.1007/s12055-025-01932-9

**Published:** 2025-03-14

**Authors:** Andreas Sarantopoulos, Ioannis Koukis

**Affiliations:** 1 Medical School, European University of Cyprus, Nicosia, Cyprus; 2 Department of Cardiac Surgery, 401 General Army Hospital, Athens, Greece

**Keywords:** Lupus aortitis, Systemic lupus erythematosus, Aortic intramural hematoma, Autoimmune vasculitis, Aortic surgery

## Abstract

Systemic lupus erythematosus (SLE) is a chronic autoimmune disorder with diverse clinical manifestations. While small vessel vasculitis is a common SLE complication, lupus aortitis is an exceedingly rare entity with limited documentation. Here, we report a novel case of lupus aortitis misleadingly appearing on imaging studies as an aortic intramural hematoma in a 68-year-old male. The patient initially exhibited fatigue, dyspnea, and pericardial effusion. Imaging studies suggested an intramural hematoma of the ascending aorta and the aortic arch, prompting urgent surgical exploration. Intraoperative findings revealed a thickened, fibrotic, and heavily calcified aorta (egg-shell aorta) without hematoma. Subsequent histopathological and immunological analyses confirmed lupus aortitis. This case underscores the diagnostic challenges posed by this rare SLE complication, which can mimic other aortic pathologies. Early recognition and individualized treatment are paramount. Further studies are needed to elucidate its pathophysiology and establish standardized management guidelines.

## Introduction

Systemic lupus erythematosus (SLE) is a chronic autoimmune multisystem disorder characterized by a highly variable presentation depending on the organs involved. The global incidence of SLE is estimated at 5.14 cases per 100,000 person-years (range 1.4–15.13) [1]. The heterogeneity of SLE arises from the diverse spectrum of antibodies and immune complexes implicated in its pathogenesis, leading to various disease subtypes with distinct prognoses, clinical manifestations, and management strategies.

Small vessel vasculitis is a relatively common manifestation of SLE, driven by immune-complex deposition in low-flow, small-caliber vessels. However, involvement of the aorta, as seen in lupus aortitis, is an exceptionally rare complication, with only a limited number of cases documented in the literature and no established treatment guidelines [2]. Notably, most reported cases of lupus aortitis are associated with life-threatening complications such as aortic dissection or aneurysm formation [3].

In this report, we present a case of lupus aortitis manifesting with radiological features that mimicked an aortic intramural hematoma on computed tomography (CT) imaging, a presentation that, to our knowledge, has not been previously reported in the literature. To verify the novelty of this presentation, we conducted a comprehensive literature review across multiple databases (PubMed/MEDLINE, Scopus, and Cochrane Library). The following Medical Subject Headings (MeSH) terms and keywords were used in various combinations: “Systemic Lupus Erythematosus,” “SLE,” “Aortitis,” “Lupus Aortitis,” “Intramural Hematoma,” and “Imaging.” This search revealed no previously published reports documenting lupus aortitis presenting as an intramural hematoma on imaging, supporting our claim that this is the first such case in the medical literature.

## Case report

A 68-year-old male patient was emergently transferred to a tertiary hospital in Athens, Greece, following a referral from a regional hospital. The patient reported a 1-month history of fatigue and exertional dyspnea, which had acutely worsened over the last week. His past medical history was notable for ischemic stroke, pericarditis, anemia, and chronic pleural and pericardial effusions of unknown origin. There was no significant family history. The patient admitted to occasional alcohol consumption but denied tobacco or recreational drug use. The rest of his medical history was unremarkable.

On arrival to our hospital, the patient was hemodynamically stable with a blood pressure of 146/87 mmHg, afebrile, and with borderline sinus tachycardia at 100 beats per minute. Pulses were palpable in all extremities and the patient was alert and oriented to time, person, and place. Mild dyspnea was noted, with a saturation of peripheral oxygen (SpO2) of 96% on room air.

Color echocardiography done at the referring hospital revealed a normal aortic root with a diameter of 36 mm, ascending aorta measuring 33 mm, and normal structure and function of the ventricles, atria, and heart valves. The ejection fraction (EF) was within normal limits, calculated at 60%. Additionally, a significant homogenous pericardial effusion was observed, with a maximum fluid thickness of 3.5 cm.

**Fig. 1:**
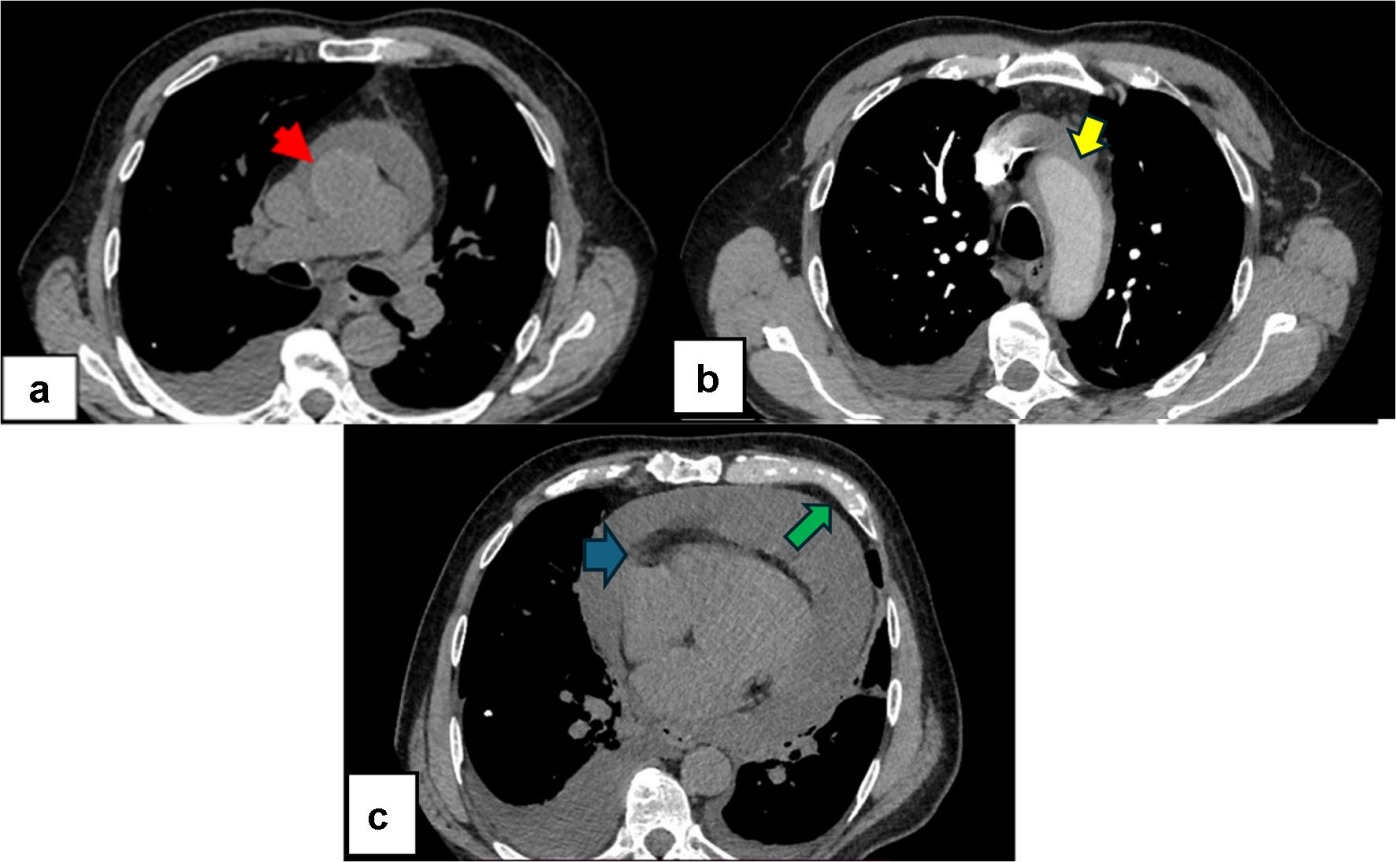
**a** Computed tomography pulmonary angiography (CTPA) scan showcasing the abnormal thickening of the ascending aortic wall, suggesting the presence of intramural hematoma (red arrow). **b** Computed tomography pulmonary angiography (CTPA) scan showcasing extent of the supposed intramural hematoma distal to the origin of the left subclavian artery (yellow arrow). **c** Computed tomography pulmonary angiography (CTPA) scan showcasing pericardial effusion (blue arrow) and pleural effusion (green arrow)

Computed tomography pulmonary angiography (CTPA) had already been performed at the referring hospital to rule out pulmonary embolism. As mentioned in the report from the referring hospital, CTPA was selected due to the patient’s tachycardia and dyspnea, raising concern for pulmonary embolism. This was ruled out, but it revealed an intramural hematoma of the ascending aorta and the aortic arch, with a maximal thickness of 7 mm. The hematoma extended from the level of the aortic root to the origin of the left subclavian artery. Findings consistent with the pericardial effusion noted on echocardiography were confirmed, showing a fluid thickness of 3.5 cm. Bilateral pleural effusions were noted. Normal-sized mediastinal lymph nodes were also visualized (Fig. 1).

Thus, without further delay, due to the life-threatening nature of this disease and since the diagnosis of aortic intramural hematoma had already been established at the referring hospital, the patient was scheduled for urgent sternotomy with a view of removing the ascending aorta and possibly the aortic arch and replacing it with a synthetic graft to treat the intramural hematoma. Of note, the patient did not mention any sudden chest or back pain.

Preoperative laboratory investigations revealed a normal white blood cell (WBC) count of 7800/mm^3^ with marked neutrophilia (83% of total WBC), anemia with a hemoglobin (Hb) level of 12.4 g/dL, and hematocrit (Hct) at 39.3%. All other laboratory parameters were within normal reference ranges.

The operation was performed under general anesthesia with the patient in the supine position. A subclavicular incision was performed, the right axillary artery was identified and incised, and an 8-mm synthetic graft was anastomosed end to side on the right axillary artery (RAA) in order to be used as the arterial cannulation site of the cardiopulmonary bypass (CPB) circuit. Next, median sternotomy was performed. The procedure commenced with dissection of the notably thickened pericardium, during which a sample was collected for biopsy. A significant pericardial fluid collection, consistent with imaging findings, was also observed. Pericardial fluid was aspirated for cytological and microbiological analyses and subsequently drained.

**Fig. 2:**
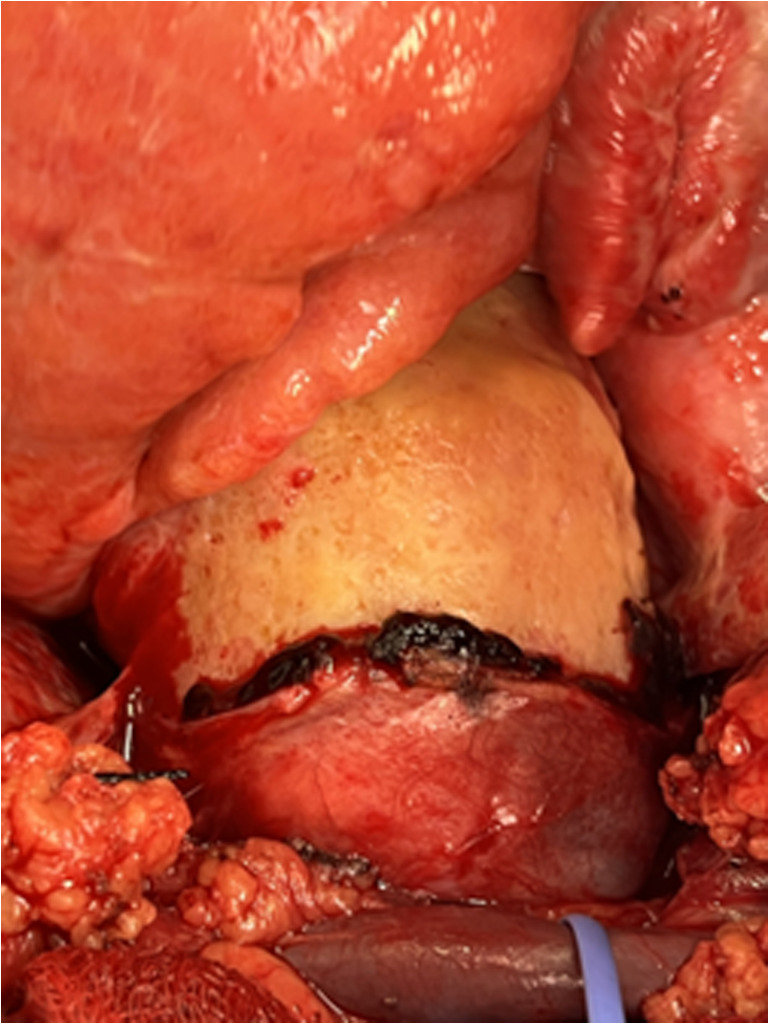
Rigid and thickened calcified casing of yellow color surrounding the aortic root and ascending aorta until just proximal to the origin of the left innominate artery

Next, the aorta was inspected which revealed a rigid, thickened, yellow-hued casing encasing the aortic root and ascending aorta, extending proximally to the origin of the left innominate artery (Fig. 2). This finding did not correlate with the initially diagnosed intramural hematoma. The rest of the ascending aorta and the aortic arch appeared normal. Thus, a decision was made not to proceed with aortic replacement since there was no apparent intramural hematoma of the ascending aorta or the aortic arch. Both pleural cavities were opened, a large amount of pleural bilateral fluid was drained, and pleural fluid was collected for additional testing.

Given the discrepancy between the imaging-based diagnosis and intraoperative findings, the surgical team elected to defer further intervention until fluid and biopsy test results became available. The RAA graft was ligated, and three chest tubes were inserted to facilitate pleural and pericardial drainage. The subclavicular incision was sutured in layers, and the sternum was closed in the usual fashion. The sternotomy wound was closed in layers.

The patient was transferred to the intensive care unit (ICU) following the operation. His postoperative course was uneventful, with no significant complications or changes in his clinical status. Chest tubes were removed on the 8th postoperative day. A color echocardiography performed on the 12th postoperative day revealed no residual pericardial fluid collection. The patient was subsequently discharged in stable condition on the 13th postoperative day. Two weeks later, a pleural effusion was detected during follow-up. The effusion was successfully drained, and no further complications were noted. Follow-up echocardiography showed no additional abnormalities.

Following the patient’s discharge, an extensive rheumatological and immune screening was conducted. The diagnosis of SLE was confirmed with significantly elevated antinuclear antibody (ANA) titers (> 1:160, homogeneous pattern) and elevated anti-double-stranded deoxyribonucleic acid (anti-dsDNA) antibody levels. Lastly, elevated levels of erythrocyte sedimentation rate (ESR) with mildly elevated levels of C-reactive protein (CRP) (ESR, 99 mm/h; CRP, 9.3 mg/L) and persistent neutrophilia with normal WBC count were inconsistent with active inflammation and further supported the diagnosis of lupus aortitis. According to European League Against Rheumatism/American College of Rheumatology (EULAR/ACR) Clinical Domains and Criteria for SLE [4], the patient had confirmed SLE diagnosis and was referred to a specialist SLE center elsewhere.

Cytological examination of the pericardial fluid was negative for malignant cells but revealed reactive mesothelial cells. Micronodular mesothelial cell hyperplasia was observed, accompanied by a significant presence of hemosiderin inclusions within the mesothelial cells. Similarly, the pleural fluid analysis was negative for malignant cells and showed a few mesothelial cell aggregates along with numerous macrophages. Lastly, histological analysis of the pericardial biopsy further revealed perivascular lymphoplasmacytic aggregates, likely representing a reactive process associated with previous episodes of pericarditis.

## Discussion

We present the first reported case of lupus aortitis mimicking the appearance of an aortic intramural hematoma on CT imaging. Intraoperative findings and subsequent histological analysis suggested aortitis without evidence of intramural hematoma (IMH). Given the rarity of this condition, there is limited literature addressing its pathophysiology, clinical characteristics, and management.

More specifically, there are no studies on the pathomechanisms of lupus aortitis. Some case reports suggested that SLE-associated serositis shared common mechanisms with lupus aortitis but the evidence provided is circumstantial, and contradictory findings have also been reported in different lupus aortitis cases [5, 6].

Similar case reports of lupus aortitis have consistently shown that patients present with non-specific constitutional symptoms, such as fatigue, fever, weight loss, and chest pain [3, 6, 7]. Consequently, the diagnosis is often made either at the time of surgical intervention [4]—as was the case in this report—or through imaging techniques such as computed tomography angiography (CTA), magnetic resonance angiography (MRA), and histological examination of tissue samples [8]. In addition, when clinical findings and imaging are incongruent, additional modalities—such as magnetic resonance imaging (MRI) spin-echo “Black Blood” sequences—can clarify the diagnosis by highlighting inflammation and ruling out IMH [9]. MRI findings, including aortic wall thickening with increased signal intensity on T2-weighted sequences, are consistent with active inflammation and were not utilized in this case.

Of note, recurrent pericardial or pleural collections of unknown origin should prompt careful evaluation for potential autoimmune or inflammatory disorders, including lupus aortitis. These effusions may be an early marker of systemic disease activity—as in our case—and could share pathogenic mechanisms with vascular inflammation. Hence, clinicians must maintain a high degree of suspicion, particularly in patients with existing SLE or unexplained serositis, as identifying and addressing occult aortitis or other vasculitides is crucial to prevent adverse outcomes.

Other conditions that can mimic IMH on imaging include acute aortic syndrome, aortoarteritis, Takayasu arteritis, giant cell arteritis (GCA), and Immunoglobulin G4 vasculitis [8]. Infectious aortitis, often caused by bacterial or fungal pathogens, can also present as a thickened aortic wall on imaging [8]. Each of these conditions has distinct clinical and laboratory features, such as systemic signs of infection in infectious aortitis or elevated inflammatory markers in Takayasu arteritis, which aid in differentiation. Accurate diagnosis requires correlating imaging findings with clinical presentation and laboratory results to avoid misdiagnosis and inappropriate treatment strategies.

Despite the growing recognition of lupus aortitis, no definitive treatment protocol has been established. Most studies recommend high-dose glucocorticoid or immunomodulatory therapy [3]. A report by Akebo et al. suggested the use of moderate-dose glucocorticoids, where prednisolone was administered at an initial dose of 0.5 mg/kg/day for 2 weeks, followed by a gradual taper, resulting in complete remission of lupus aortitis [2]. Furthermore, patients with lupus aortitis complicated by aortic aneurysm or dissection may benefit from surgical or combined surgical and glucocorticoid treatment approaches [10]. However, treatment outcomes have shown considerable heterogeneity, and the absence of randomized controlled trials prevents the formulation of a standardized treatment approach.

## Conclusion

This case underscores the diagnostic challenge of distinguishing lupus aortitis from other acute aortic pathologies. Comprehensive imaging, including advanced modalities such as MRI, intraoperative assessment, and histological confirmation, are critical for accurate diagnosis and appropriate management. The overlap in imaging findings between lupus aortitis and other conditions such as intramural hematomas or aortoarteritis highlights the importance of a multidisciplinary approach to patient care. By deferring unnecessary surgical intervention, this case highlights the value of careful intraoperative assessment and tissue sampling. Given the rarity of this condition, early recognition and individualized treatment plans, based on the available literature, are essential to manage this rare but potentially fatal SLE complication.

In this case, the absence of an intramural hematoma during intraoperative inspection prompted the surgical team to forgo unnecessary aortic replacement, emphasizing the importance of direct visualization and histological analysis in decision-making. This approach not only avoided unwarranted surgical risks but also highlighted the diagnostic complexities associated with lupus aortitis. Further research is needed to elaborate on the mechanisms, risk factors, and optimal treatment targets for lupus aortitis to guide clinicians in managing such complex cases.

## Data Availability

Not applicable.
